# Integrative analysis of Poly(A)-seq and RNA-seq reveals transcriptional regulation of poly(A) tail length in tuberculosis

**DOI:** 10.1128/spectrum.02825-24

**Published:** 2026-02-27

**Authors:** Bahetibieke Tuohetaerbaike, Jie Wang, Ying Li, Liya Yue, Peihan Wang, Cuidan Li, Hao Wen, Wenbao Zhang, Jing Wang, Fei Chen, Xin Hu

**Affiliations:** 1 State Key Laboratory of Pathogenesis, Prevention and Treatment of High Incidence Diseases in Central Asia, Xinjiang Medical University 74790, Urumqi, Xinjiang, China; 2 National Genomics Data Center, China National Center for Bioinformation 74696 , Beijing, China; 3 Beijing Institute of Genomics, Chinese Academy of Sciences 74696 , Beijing, China; 4 University of Chinese Academy of Sciences, Beijing, China; 5 The First Affiliated Hospital of Xinjiang Medical University 159427, Urumqi, China; 6 Department of Respiratory Medicine, First Affiliated Hospital of Xinjiang Medical University 159427, Urumqi, Xinjiang, China; 7 Department of Respiratory Medicine, Second Affiliated Hospital of Hainan Medical University Haikou 614533, Hainan, China; 8 NHC Key Laboratory of Tropical Disease Control, Hainan Medical University 12455, Haikou, China; 9 Key Laboratory of Viral Pathogenesis & Infection Prevention and Control (Jinan University), Ministry of Education 47885, Guangzhou, China; Shenzhen University School of Medicine, Shenzhen, China

**Keywords:** tuberculosis, Poly(A)-seq, poly(A) tail length, post-transcriptional regulation, RNA-seq

## Abstract

Tuberculosis (TB) is a serious global health problem, and the molecular mechanisms underlying TB pathogenesis remain poorly understood to date. Differential expression of many genes is an important contributor to TB, and poly(A) tails have previously unrecognized regulatory roles in this process that may impact gene expression. In this study, we combined Poly(A)-seq and RNA-seq to preliminarily investigate the link between poly(A) tail length and gene expression regulation in TB. The results indicated that the majority of genes harbor a longer poly(A) tail in TB, and the transcript levels of these genes were likely to be precisely regulated by poly(A) tail length. Notably, we identified 22 immune/inflammation-related genes that exhibited significant variations in both poly(A) tail length and expression levels. Furthermore, we focused on CHI3L1, associated with a robust antibacterial response, and experimentally validated its poly(A) tail length and expression level, showing that alterations in CHI3L1 poly(A) tail length affect gene expression, which may be important for *Mycobacterium tuberculosis* infection. In conclusion, this study generates hypotheses about post-transcriptional regulation in TB granulomas and may contribute potential strategies and ideas for future TB control.

**IMPORTANCE:**

Tuberculosis (TB), caused by *Mycobacterium tuberculosis* (Mtb), remains a global health challenge, with granulomas—its defining histopathological feature—playing a crucial role in the host’s defense and patient outcomes. While poly(A) tail processing, a critical post-transcriptional regulator of RNA lifecycle events, has been studied in diseases like repeat expansion disorders, its role in TB pathogenesis remains unexplored. This study is the first to integrate Poly(A)-seq and RNA-seq to investigate the significance of poly(A) tail length regulation in tuberculosis granulomas. Our findings provide new insights into Mtb pathogenesis and the human immune response, and offer promising avenues for developing targeted host-directed therapeutic strategies.

## INTRODUCTION

Tuberculosis (TB), a communicable disease caused by *Mycobacterium tuberculosis* (Mtb), poses a serious threat to human health as a major public health problem worldwide (1). According to the World Health Organization Global TB Report 2023, there were more than 10.6 million people newly diagnosed with TB, and about 1.3 million deaths globally in 2022. In China, there were 748,000 new cases and 30,000 deaths, ranking no. 3 among the 30 high-burden countries for tuberculosis (2). Moreover, the outbreak of COVID-19 has reversed the progress made in combating TB over the past decade, leading to an increasing trend in TB incidence. The development of new treatments for TB remains an urgent priority.

Granulomas are the hallmark pathological structures of TB and form the critical pathogen-host interface during Mtb infection. They serve to “wall off” the mycobacteria, which not only aids in the survival of the mycobacteria but also acts as the host’s defense response against tuberculosis infection (3). In the early stages of granuloma formation, they allow bacteria to transfer to newly arrived macrophages, thereby facilitating the spread of infection. As adaptive immunity develops, the granuloma serves to inhibit bacterial growth. However, under some circumstances, the macrophages within the infected granuloma may undergo necrosis, creating a progressively hypoxic environment and the formation of an acellular core called caseum, which supports bacterial growth and facilitates transmission to the next host (4, 5). Therefore, the interaction between Mtb and the host cells it encounters is crucially important in determining the outcome of disease progression.

As we know, poly(A) tails are a dynamic and important modification of RNA, serving as a binding site for poly(A)-binding proteins (PABPs), which control multiple events throughout the RNA lifecycle, including nuclear export, translation, and mRNA stability (6). The dynamic nature of poly(A) tails, therefore, is vital in almost every aspect of eukaryotic biology, including in early development, the inflammatory response, and synaptic plasticity (7). In general, the longer the poly(A) tail, the more efficiently the mRNA is translated. It has been reported that poly(A) tail processing has an important role in repeat expansion diseases and promises great potential as a candidate therapeutic target (8). Therefore, understanding the role of poly(A) tail in gene expression regulation and the relationship between poly(A) tail length and translation efficiency is helpful to improve the complete understanding of the biological function of poly(A) tail. It is worth noting that there is already a study reporting that during the activation of macrophages, over 1,500 types of mRNA, including immune-related genes, pro-inflammatory genes, and post-transcriptional regulatory proteins, experienced significant changes in tail length (9). Macrophages are the primary host cells for Mtb and are crucial for the early clearance of the pathogen. This suggests that post-transcriptional regulation of poly(A) tails may play a significant role in the pathogenesis of tuberculosis. However, there are currently no studies on the role of poly(A) tails in the pathogenic mechanisms of tuberculosis.

To investigate the critical role of poly(A) tail in the pathogenesis of TB and to reveal its dynamic regulation of gene expression in granulomas, we employed Poly(A)-seq and RNA-seq on lung granuloma tissues from TB patients, preliminarily constructed a detailed dynamic map of poly(A) tail length in relation to mRNA expression, and revealed the key roles of post-transcriptional regulation of poly(A) tails in immune response, ion transport, and signal transduction in TB lung granulomas. Additionally, we identified 22 genes with significant changes in both poly(A) tail length and expression levels that may serve as potential candidate therapeutic targets for the development of host-directed new immunotherapies against TB.

## MATERIALS AND METHODS

### Sample acquisition

All subjects underwent wedge resection or intra-operative biopsy of their lung lesions for frozen section pathologic analysis to confirm pulmonary tuberculosis prior to lobectomy. Three suspected TB patients who received lobectomy were included in our study, and postoperative pathological findings were pulmonary tuberculosis. All samples were obtained by swabbing lung tissue in the operating room. Here, all patients with granuloma and fibrotic/caseous TB lesions in the lungs were referred for the surgical management of pulmonary TB. For each TB patient, immediately after surgery, samples of lung tissue (10 g–15 g) were collected simultaneously from the tuberculoma wall and from the lung tissue about 5 cm away from the tuberculoma.

### RNA extraction and sequencing

Total RNAs were first extracted from tissues according to the standard Trizol method and then treated with RQ1 DNase (Promega) to remove DNA. The quality and quantity of the purified RNA were determined by measuring the absorbance at 260 nm/280 nm (A260/A280) using Nanodrop One (Thermo). RNA integrity was further verified by 1.5% agarose gel electrophoresis.

For each sample, 1 µg of total RNA was used for RNA-seq library preparation. Ribosomal RNAs were depleted with the Ribo-Zero Gold rRNA Removal Kit (Vazyme, N406-01). The purified RNAs were used for RNA-seq library preparation with the KAPA Stranded mRNA-Seq Kit for Illumina Platforms (KK8544). They were fragmented and then converted into double-strand cDNA. Following end repair and A-tailing, the DNAs were ligated to diluted Roche Adaptor (KK8726). After purification, the fragments (300–500 bp) were amplified, purified, quantified, and stored at −80°C before sequencing. The strand marked with dUTP (the second cDNA strand) was not amplified, allowing strand-specific sequencing. For high-throughput sequencing, the libraries were prepared according to the manufacturer’s instructions and sequenced on Illumina NovaSeq 6000 system.

### RNA-seq raw data preprocessing

Raw reads containing more than 2-N bases were first discarded. Then adaptors and low-quality bases were trimmed from raw sequencing reads using FASTX-Toolkit (version 0.0.13). Short reads less than 16 nt were also dropped. After that, clean reads were aligned to the GRCh38 genome by Tophat2 (10) allowing four mismatches. Uniquely mapped reads were used for gene read number counting and FPKM calculation (fragments per kilobase of transcript per million fragments mapped) (11).

### Poly(A)-seq library generation and sequencing

To systematically analyze the poly(A) tail profile, we performed Poly(A)-seq as previously reported (12). For each sample, 5 µg of total RNAs was used for Poly(A)-seq library preparation. Total RNAs were then digested by RNase T1 (Thermo, EN0541), and polyadenylated mRNAs were purified and concentrated with oligo(dT)-conjugated magnetic beads (Vazyme, N401-01) before Poly(A)-seq library preparation, followed by addition of 3′ adaptor ligation (Gnomegen, K02420-L). Reverse transcription was performed with Terminal-Tagging oligo cDNA (complemented with 3′ adaptor) using the SMARTer Stranded RNA-Seq Kit (TAKARA, 634837). The synthesized cDNAs were further purified and amplified, and the PCR products (200 bp–500 bp) were purified, quantified, and stored at −80°C until sequencing. For high-throughput sequencing, the Illumina NovaSeq 6000 system was still used for 150 nt paired-end sequencing.

### Poly(A)-seq data processing

The Poly(A)-seq data were processed using the paFinder pipeline which was previously reported (12). In detail, after trimming off poly(G) and 5′, 3′ adaptor sequences from Poly(A)-seq raw reads, we first searched for the poly(A) regions in the reads. We began the search by scanning the full reads and finding the first 9A sequence (allowing 0.1 error rate) closest to the 5′ end and 6A (allowing 0.2 error rate) closest to the 3′ end. The sequence in between was extracted as the preliminary poly(A) region, and reads without preliminary poly(A) region or with preliminary poly(A) <10 nt were discarded. After extracting the preliminary poly(A) region, the remaining sequence toward the 5′ end of the reads was used for mapping to reference human genome (GRCh38). In the mapping step, at least 20 nt was required, and Tophat2 was used allowing up to two mismatches. Reads that were uniquely mapped to the human genome were kept for analysis of poly(A) length. Next, we scanned the preliminary poly(A) region to label any consecutive non-A sequences ≥5 nt in length. The longest sequence within the region not containing consecutive non-A’s was determined as the poly(A) region, and the length was counted. Student’s *t*-test was used to compare poly(A) tail length between two groups. *P*-values <0.05 were set as the cut-off criteria for identifying genes with significantly differential poly(A) tail length.

### Spike-in RNA preparation

DNA oligos containing a 40-nt poly(A) sequence (Tianyi Huiyuan Inc., Wuhan) and a 120-nt poly(A) sequence (IDT, Singapore) were chemically synthesized to generate poly(A)-tail-length standards.

The 40-nt poly(A) sequence is located between the 5′ end adaptor sequence (5′-TAATACGACTCACTATAGGGTTTAACGCGAATTAATTCTGTGGAATGTGTGTCAGTTAGG-3′) and the 3′ end adaptor sequence (5′-CATTGCCTAGAGTCGGACTGA-3′). The 5′ end adaptor sequence contained the T7 promoter sequence and a segment of PcDNA3.1 plasmid sequence, and the 3′ end adaptor sequence contained the sequence of BsrD1 cutting sites.

The 120-nt poly(A) sequence is located between the 5′ end adaptor sequence (5′-TAATACGACTCACTATAGGGTCGACGCTCAAGTCAGAGGTGGCGAAACCCGACAGGACTA-3′) and the 3′ end adaptor sequence (5′-CATTGCCTAGAGTCGGACTGA-3′). The 5′ end adaptor sequence contained a 20-nt T7 promoter sequence, which was followed by a 40-nt or 39-nt PcDNA3.1 plasmid sequence, and the 3′ end adaptor sequence contained a BsrD1 cutting site allowing the complete removal of the 3′ adaptor sequence prior to *in vitro* transcription.

The DNA templates for *in vitro* synthesis of the spike-in poly(A) tail RNA were amplified using the forward primer containing the T7 promoter sequence (5′-TAATACGACTCACTATAGGG-3′) and the reverse primer targeting 3′ end adaptor sequence (5′-TCAGTCCGACTCTAGGCA-3′). The PCR products were purified by VAHTS DNA Clean Beads (Vazyme, N411-03) and then cut with BsrD1 (NEB, R0574S) to remove the 3′ end adaptor sequences, leaving the 3′ poly(A) tail sequence at the very end of the template. The enzyme-digested product was gel purified using a Qiagen column kit after electrophoresis on a 4.0% agarose gel. The *in vitro* transcription was carried out using TranscriptAid T7 High Yield Transcription Kit (Thermo, K0441). The resulting 80-nt spike-in RNA (40-nt adaptor followed by 40-nt poly(A) tail) and 160-nt spike-in RNA (40-nt adaptor followed by 120-nt poly(A) tail) were purified on a 12% denaturing urea polyacrylamide gel.

### Differentially expressed genes (DEGs) and poly(A) length differential genes analysis

The R Bioconductor package edgeR (13) was utilized to screen for DEGs from RNA-seq data. A false discovery rate (FDR) <0.05 and fold change (FC) ≥ 2 or ≤ 0.05 were set as the cut-off criteria for identifying DEGs. For Poly(A)-seq data, the Wilcoxon test was used to analyze the difference in the median value of poly(A) length, and FC ≥ 1.5 or ≤ 2/3 and *P*-value <0.05 were used as the threshold to filter the genes with a significant difference in length change.

### Functional enrichment analysis

To explore functional categories of differential genes, Gene Ontology (GO) terms and Kyoto Encyclopedia of Genes and Genomes (KEGG) pathways were identified using the KOBAS 2.0 server (14). The hypergeometric test and Benjamini-Hochberg FDR controlling procedure were used to identify the enriched terms.

### RT-qPCR validation

Total RNA remaining from RNA-seq library preparation was used for qPCR. RNA was reverse transcribed into cDNA using M-MLV Reverse Transcriptase (Vazyme). Real-time PCR was performed with the StepOne Real-Time PCR System using the Hieff qPCR SYBR Green Master Mix (Low Rox Plus; YEASEN, China). The PCR conditions consist of denaturing at 95°C for 5 min, 40 cycles of denaturing at 95°C for 15 s, and annealing and extension at 60°C for 30 s. PCR amplifications were performed in triplicate for each sample.

### Poly(A) tail length measurement

Poly(A) tail length was detected by PCR-based amplification using an NGS library. PCR amplification was performed using two primer sets: (i) a gene-specific forward and reverse primer set designed upstream of the polyadenylation site as a control, and (ii) a gene-specific forward primer and the universal reverse primer, which is provided with the Balancer NGS Library Preparation Kit for small/microRNA (K02420-S), to generate a product including poly(A) length. PCR products were detected by 2% agarose gel electrophoresis.

### Other statistical analysis

Principal component analysis (PCA) was performed using the R package factoextra (https://cloud.r-project.org/package=factoextra) to show the clustering of samples with the first two components. The pheatmap package (https://cran.r-project.org/web/packages/pheatmap/index.html) was used to perform clustering based on Euclidean distance.

## RESULTS

### Clinical characteristics of patients

In this study, three TB patients were enrolled to explore the association between poly(A) tail length and its transcriptional regulation in pulmonary tuberculosis. Samples of pathological lung granulomas and adjacent normal lung tissue (located 5 cm away from the tuberculoma) were simultaneously collected from each patient, with distinct histological lesions (Fig. S1–S3). The clinical characteristics of the TB patients were shown in Table S1.

### Comparison of poly(A) tail length between tuberculous granuloma and normal lung tissue

**Fig 1:**
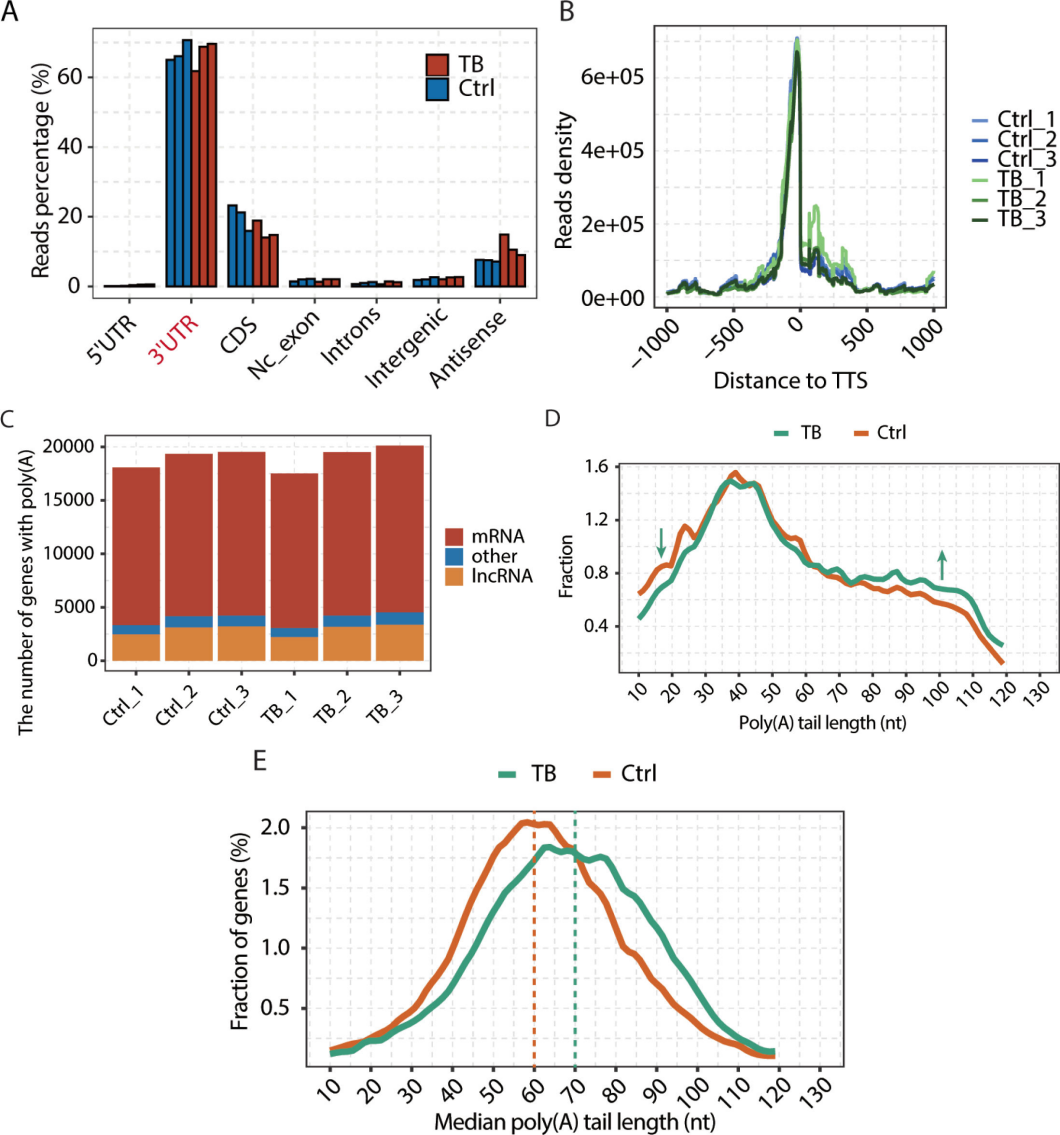
Distribution of poly(A) tails between tuberculosis granuloma samples and normal lung tissue samples. (**A**) Poly(A) reads distribution of granuloma and Ctrl samples in different genomic regions. (**B**) Line plot showing the distribution of poly(A) reads around the TTS (±1 kb). (**C**) The number of different types of genes with poly(A)-containing reads. (**D**) Distribution of different length of poly(A) tails in granuloma and Ctrl samples. The x-axis indicates the length of poly(A) tails, and the y-axis indicates the fractions of different length of poly(A) tails in granuloma and Ctrl samples. (**E**) Distribution of median poly(A) tail lengths for all genes. The x-axis indicates the median length of poly(A) tails for all genes, and the y-axis indicates the gene fractions with different poly(A) tail lengths in the granuloma and Ctrl samples.

To evaluate the regulatory role of poly(A) tails, we performed Poly(A)-seq analysis on pulmonary tuberculosis granuloma (MTB) and adjacent normal (Ctrl) lung tissue samples from three patients. The vast majority of reads in Poly(A)-seq mapped to the 3′ UTR region (Fig. 1A), responsible for the 3′ end formation of most genes in relation to transcription termination, and additionally revealed a preferential localization at transcription termination sites (TTS) (Fig. 1B), implying a tight association with termination of transcription in defining the 3′ ends of genes. Notably, although the majority of poly(A) tails belonged to mRNAs, approximately 15% were also found to be long non-coding RNAs and some other types of genes (Fig. 1C). The distribution of poly(A) tails of different lengths (Fig. 1D) and median tail lengths (Fig. 1E) in different samples was subsequently examined, and it was found that more reads with longer poly(A) tails were detected in tuberculosis granuloma samples, and the poly(A) tail lengths were generally longer than those of normal lung tissue, suggesting that poly(A) may play an important regulatory role in resistance to Mtb infection.

**Fig 2:**
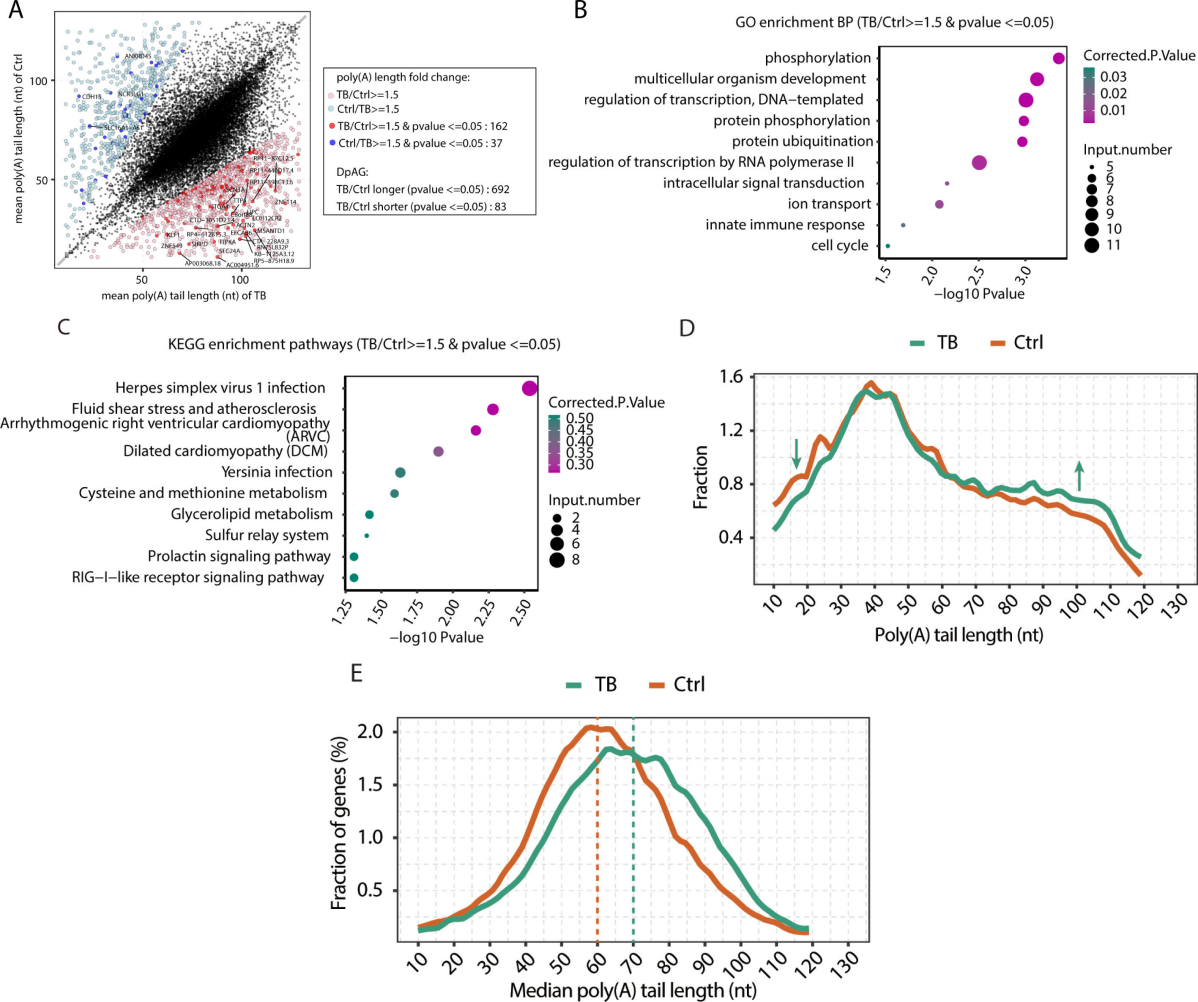
Dynamic changes of poly(A) tail length between tuberculous granulomas and normal lung tissues. (**A**) Scatter plot showing the mean median poly(A) length of individual genes in MTB and Ctrl samples. Genes with significant changes in their median tail length (DPGs) between MTB and Ctrl samples (absolute fold change ≥1.5 and *P*-value ≤0.05 based on Student’s *t*-test) are denoted as colored dots. Red dots represent genes with shortened poly(A) tail in Ctrl, and blue dots indicate genes with shortened poly(A) tail in MTB. (**B**) Top 10 GO biological process terms enriched by genes with longer poly(A) tail with fold change ≥1.5 and *P*-value ≤0.05 between MTB and Ctrl samples. (**C**) Top 10 KEGG pathways enriched by genes with longer poly(A) tail with fold change ≥1.5 and *P*-value ≤0.05 between MTB and Ctrl samples. (**D**) Distribution of different lengths of poly(A) tails in granuloma and Ctrl samples. The *x*-axis indicates the length of poly(A) tails, and the *y*-axis indicates the fractions of different lengths of poly(A) tails in granuloma and Ctrl samples. (**E**) Distribution of median poly(A) tail lengths for all genes. The *x*-axis indicates the median length of poly(A) tails for all genes, and the *y*-axis indicates the gene fractions with different poly(A) tail lengths in the granuloma and Ctrl samples.

We then compared and identified differential genes with significant alterations in mean poly(A) tail length between the MTB and Ctrl samples, indicating that 692 genes in the MTB group had significantly longer poly(A) tails than in the Ctrl group, and only 83 genes had significantly shorter poly(A) tails, with 199 of these genes exhibiting high fold changes, including 162 genes with longer poly(A) tails and 37 genes with shorter poly(A) tails (Fig. 2A). This observation reinforces the trend of a higher prevalence of genes with extended poly(A) tails in TB samples compared to Ctrl samples.

Furthermore, GO and KEGG analysis of genes with significantly elongated poly(A) tails (FC ≥ 1.5, *P*-value <0.05) in TB granulomas showed a significant enrichment in protein post-translational modifications (including “phosphorylation,” “protein phosphorylation,” “protein ubiquitination”), regulation of gene expression (e.g., “multicellular organism development,” “regulation of transcription, DNA-templated,” “regulation of transcription by RNA polymerase II”), as well as pathways related to the “innate immune response,” “intracellular signal transduction,” and “ion transport” (Fig. 2B and C). Notably, phosphorylation is a major regulatory mechanism in Mtb and plays key roles in bacterial growth and survival (15). Meanwhile, dysregulation of the innate immune response during Mtb infection is also a major contributor to disease progression (16). These findings suggest that Mtb infection might lead to alterations in poly(A) tail length of phosphorylated or immune-related genes, thereby affecting the stability and translational efficiency of these mRNAs, which in turn causes an imbalance in host defense mechanisms and reduces resistance to TB.

### Transcriptomic profiles between granuloma and Ctrl samples

**Fig 3:**
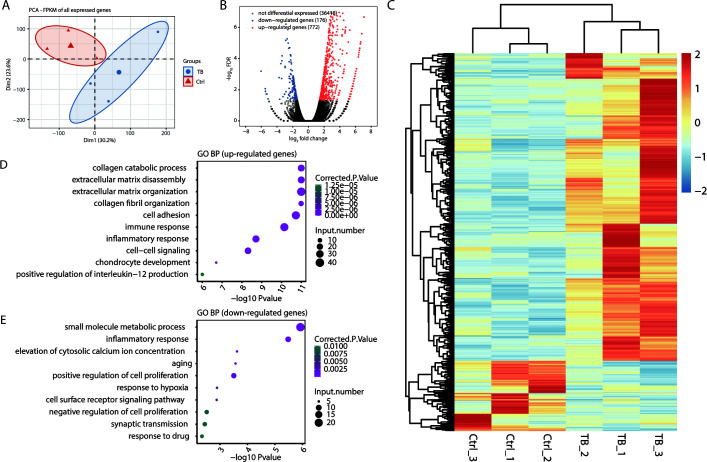
Transcriptome profiling and alteration of gene expression between tuberculous granulomas and normal lung tissues. (**A**) PCA showing differentially expressed profiles for all expressed genes between the TB and Ctrl samples. (**B**) Volcano plot showing significantly upregulated (red dots) and downregulated (blue dots) genes between the two groups. (**C**) Heatmap showing DEGs among TB and Ctrl samples. (**D**) The top 10 representative GO biological processes of upregulated genes. (**E**) The top 10 representative GO biological processes of downregulated genes.

We further performed RNA-seq on both the TB granulomas and adjacent normal lung tissue samples. PCA revealed distinct expression profiles between MTB and control samples (Fig. 3A). Furthermore, a total of 948 DEGs were identified, with 772 upregulated and 176 downregulated in TB samples (Fig. 3B and C), suggesting that Mtb is likely to function primarily by causing upregulation of expression of numerous genes in lung tissue.

GO analysis indicated that upregulated genes in TB primarily engaged in ECM remodeling (including collagen catabolic processes, extracellular matrix disassembly, organization, collagen fibril organization, and cell adhesion), immune/inflammatory responses, and cell-cell signaling (Fig. 3D). Conversely, downregulated genes focused on metabolic functions (such as small molecule metabolism and hypoxia response), cellular signaling (including calcium ion concentration regulation and receptor signaling), and cell growth regulation (aging and cell proliferation modulation) (Fig. 3E). These findings underscore their significant contribution to the host’s defense mechanisms against Mtb infection.

### Association between poly(A) tail length and transcriptional regulation in pulmonary tuberculosis granuloma

**Fig 4:**
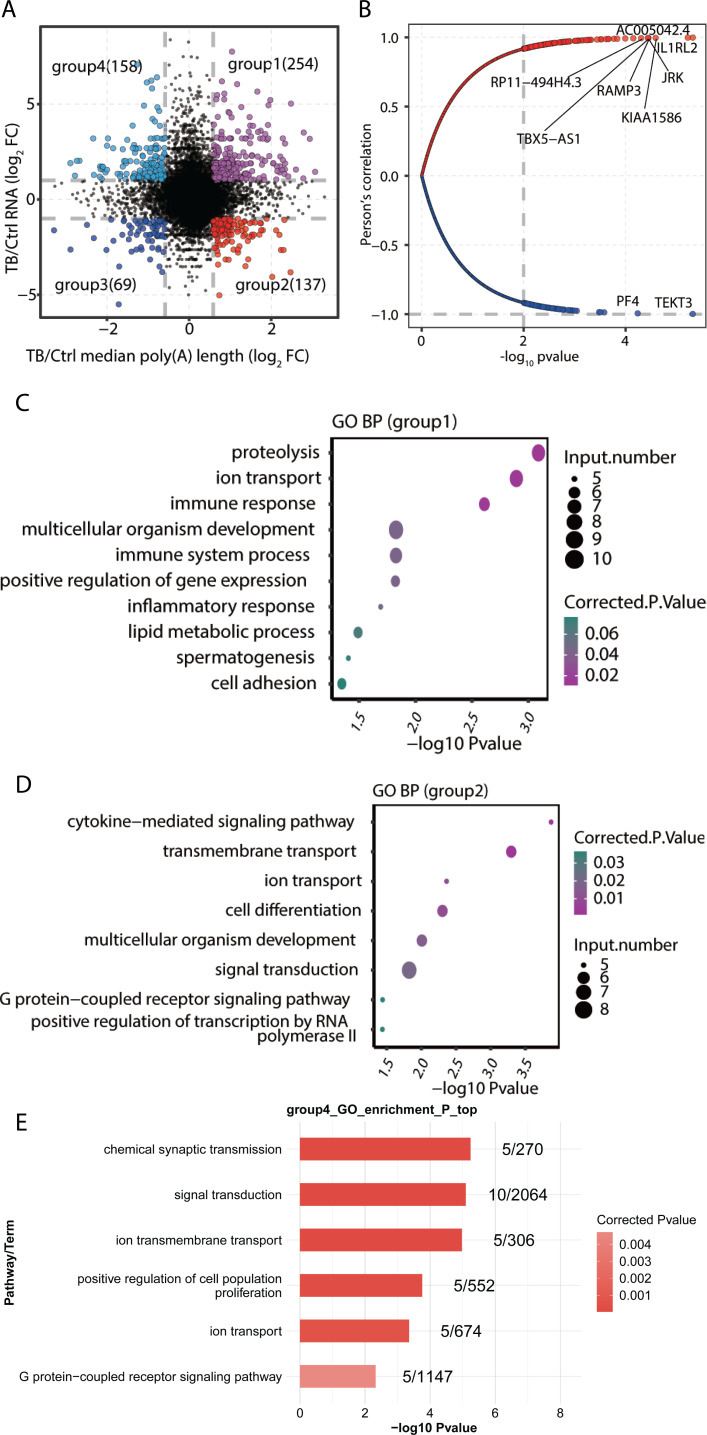
Association of gene transcriptional expression level and poly(A) tail length in TB granuloma samples. (**A**) Scatter plots illustrate the relationship between changes in RNA abundance (TB/Ctrl) for each gene (*y*-axis) and alterations in poly(A) tail length (*x*-axis). Genes with a poly(A) length FC ≥ 1.5 and an absolute DEG fold change (|FC|) ≥ 2 in TB granuloma and control samples are marked with red dots. Conversely, genes with a poly(A) tail length FC ≤ 2/3 and DEG |FC| ≥ 2 in TB granuloma and control samples are highlighted with blue dots. (**B**) Pearson correlation coefficient between gene expression level and poly(A) tail length. Genes with significant correlations (*P* < 0.01) are denoted as larger dots. Genes with *P* < 0.0001 are labeled on the figure. (**C–E**) Functional pathway analysis of genes in group 1 to group 4.

To investigate the influence of poly(A) tail length on gene transcription levels, we conducted an in-depth investigation into the dynamics between poly(A) tail length variations and alterations in gene-specific RNA abundance across both MTB and Ctrl samples. Based on the differential genes in Poly(A)-seq and RNA-seq, we categorized them into four distinct groups: groups 1 and 2 consisted of 254 genes and 137 genes, respectively, which showed significant upregulation and downregulation corresponding to an increase in poly(A) tail length. Conversely, groups 3 and 4 included 69 genes and 158 genes, respectively, which were significantly downregulated and upregulated as the poly(A) tail length decreased (Fig. 4A). Subsequently, these observations were reinforced by Pearson correlation analysis, revealing a robust correlation between gene expression levels and poly(A) tail lengths, with most genes showing positive correlations (Fig. 4B). Our results imply that poly(A) tail length serves as a critical regulator in the intricate machinery of gene expression.

GO analysis of genes with a positive correlation to poly(A) tail length indicated that 254 upregulated genes (group 1) were significantly enriched in proteolysis, ion transport, immune response, multicellular organism development, immune system processes, positive regulation of gene expression, and inflammatory response, suggesting an active immune activation environment and a positive regulatory effect of extended poly(A) tails on the gene expression associated with these functions within TB granulomas (Fig. 4C). Conversely, GO analysis of 137 genes negatively correlated with poly(A) tail length (group 2) revealed significant enrichment in the cytokine-mediated signaling pathway, hinting that longer poly(A) tails might inhibit cytokine expression in TB patients. Additionally, processes such as transmembrane transport, ion transport, cell differentiation, multicellular organism development, signal transduction, G protein-coupled receptor signaling pathway, and positive regulation of transcription by RNA polymerase II were also significantly enriched, some of which were similarly enriched in group 4, like signal transduction, ion transport, and G protein-coupled receptor signaling pathway, etc (Fig. 4D and E). Given that gene expression levels in both group 2 and group 4 were negatively associated with poly(A) tail length, these findings imply that the gene expression dysregulation observed in TB granulomas, particularly in signal transduction and ion transport, is likely attributable to changes in poly(A) tail length.

### Identification of genes with significant changes in both poly(A) tail length and expression levels

**Fig 5:**
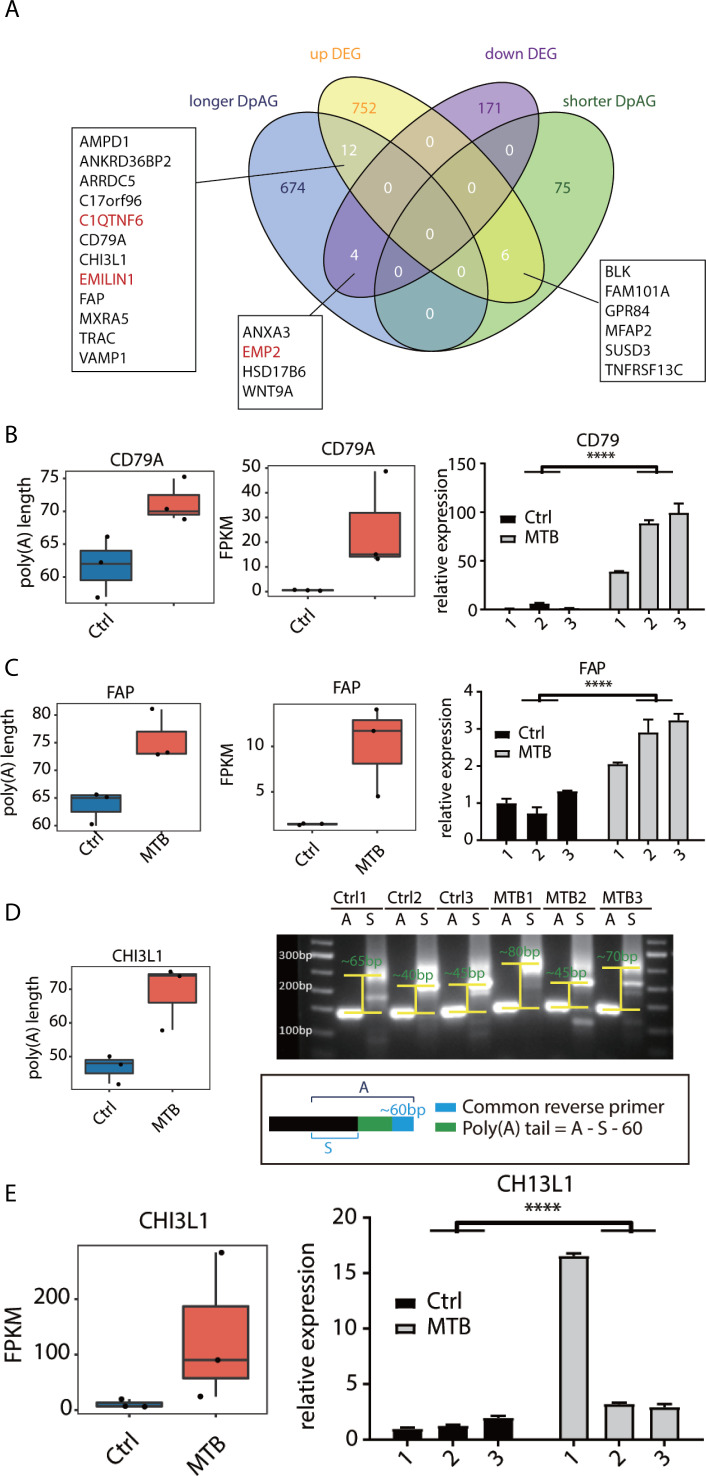
Identification of genes with significant changes in both poly(A) tail length and expression levels. (**A**) Venn diagram showing the overlap of DEGs (FDR ≤ 0.05) and DpAGs (*P*-value ≤0.05). (**B**) Poly(A) tail length (left panel) and expression level (middle panel) of CD79A. RT-qPCR was performed to validate the expression. (**C**) Poly(A) tail length (left panel) and expression level (middle panel) of FAP. RT-qPCR was performed to validate the expression. (**D**) Poly(A) tail length distribution (left panel) of CHI3L1. Poly(A) tail length of CHI3L1 was measured by PCR-based method (Hire-PAT) in MTB and Ctrl samples. Gene-specific forward and reverse primers (S) amplified the region just upstream of poly(A) tails of target genes (right-bottom panel). (**E**) Expression level of CHI3L1 was validated using RT-qPCR. ****P < 0.0001.

We further screened the genes that both displayed significant changes in poly(A) tail length and expression levels, and after overlapping, we identified 12 genes that were significantly upregulated with increases in poly(A) length (AMPD1, ANKRD36BP2, ARRDC5, C17orf96, C1QTNF6, CD79A, CHI3L1, EMILIN1, FAP, MXRA5, TRAC, VAMP1), among which six were immunometabolism-related genes, suggesting a positive regulatory role of increased poly(A) tail length on the expression of immune-related genes. Conversely, four genes (ANXA3, EMP2, HSP17B6, WNT9A) were significantly downregulated with longer poly(A) tails. Additionally, six genes (BLK, FAM101A, GPR84, MFAP2, SUSD3, TNFRSF13C) showed significant upregulation as poly(A) tail lengths decreased, including three immune-related and two cell adhesion-related genes, indicating diverse impacts of poly(A) tail modifications on gene expression (Fig. 5A; Table S2). Next, we also performed RT-qPCR experiments to verify the expression of some genes involved in immunity or apoptosis, such as CD79A (Fig. 5B) and FAP (Fig. 5C), and the results were consistent with expectations. Importantly, we double-validated the poly(A) tail length and expression of CHI3L1 and showed that the poly(A) tail length of CHI3L1 in the MTB group was indeed increased, and the expression level was also significantly higher compared to the Ctrl group (Fig. 5D and E). This result suggests that the expression changes of these genes were likely to be regulated by the poly(A) tail lengths, and this regulatory mechanism probably plays a crucial role in Mtb infection.

## DISCUSSION

Eukaryotic mRNA 3′ end formation is a key step in the production of mature transcripts and is important in gene regulation. Besides UTRs, poly(A) tail length is another crucial factor in protein synthesis, influencing mRNA stability and translation efficiency (17). Our study pioneers the exploration of how poly(A) tail length variations correlate with gene expression in pulmonary tuberculosis granuloma samples. We found significant links between transcript abundance and poly(A) tail length changes, with the tails being notably longer in the tuberculosis samples compared to normal lung tissue.

In this study, we discovered significant elongation of poly(A) tails in immune-related genes within tuberculosis granulomas, accompanied by a notable increase in their expression levels. This enhancement is due to mRNAs with extended tails more readily binding Poly(A) Binding Protein (PABPC) (6), which interacts with translation initiation factor eIF4G and termination factor eRF3, enhancing translation efficiency and stability (18). A previous study has also shown that the activation of macrophages, the primary host cells for tuberculosis bacteria in humans, is dynamically and extensively influenced by the length of the polyadenylate tail (9), providing strong support for our findings. These findings demonstrate the critical role of post-transcriptional regulatory mechanisms in the host’s immune responses, which require rapid and adaptable gene regulation to combat pathogenic challenges.

Overall, our findings highlight the crucial role of the complex immune metabolic environment within granulomas in combating pathogens and emphasize the importance of post-transcriptional regulation in this process. It extends previous research that primarily focused on changes in molecular abundance at the transcriptional, protein, or metabolic levels within granulomas. This insight not only provides a new understanding of the intricate immune mechanisms within tuberculosis granulomas but also underscores the significance of post-transcriptional regulation in developing innovative treatments for tuberculosis.

Additionally, genes related to signal transduction and ion transport within tuberculosis granulomas show significant changes in both poly(A) tail length and expression levels. Previous research has reported that Mtb requires micronutrients like metal ions for survival and pathogenicity within the host system (19). Our findings further reveal that these biological processes are regulated by the length of the poly(A) tails. Furthermore, Mtb can utilize host-derived fatty acids, lipids, and cholesterol as alternative carbon sources during infection through pathways mediated by G protein-coupled receptors (20, 21). In our study, we noted changes in both the poly(A) tail length and transcriptional levels of genes involved in these pathways, which underscores a substantial post-transcriptional regulatory effect. This research is pioneering in showing that the regulation of poly(A) tail length plays a crucial role in the bacterial adaptation to the host environment and in promoting granuloma formation, indicating the potential for developing therapeutic candidate strategies targeting the poly(A) tail.

We also identified 22 candidate genes that exhibited significant changes in both poly(A) tail length and expression levels. Such alterations could impact the synthesis and functionality of the proteins encoded by these genes. Among these, nine genes (CD79A, CHI3L1, MXRA5, FAP, C1QTNF6, TRAC, BLK, TNFRSF13C, GPR84) are crucial for immune functions, including maintaining immune balance, promoting inflammatory responses, and facilitating tissue repair. Particularly, CD79A, TRAC, BLK, and TNFRSF13C are highly expressed in granuloma B or T cells, playing roles in antigen presentation, B cell receptor signaling, and the maturation, differentiation, and survival of B and T cells (22–27); CHI3L1, MXRA5, FAP, C1QTNF6, and GPR84 are crucial in activating inflammatory factors, immune responses, and tissue fibrosis (28–32). Six other genes (VAMP1, EMP2, MFAP2, EMILIN1, SUSD3, ANXA3) are related to cell adhesion and signal transduction. Specifically, VAMP1 and EMP2 facilitate vesicle transport and synaptic signaling (33, 34); MFAP2, EMILIN1, and SUSD3 regulate cell adhesion and migration (35–38); ANXA3 mediates calcium-dependent phagosome-lysosome fusion to support the bactericidal activity of neutrophils (39). These cell adhesion biological processes induce signaling changes that can regulate the opening state of ion channels. In turn, changes in ion transport can regulate cell adhesion and migration by affecting the activity of intracellular signaling molecules. Additionally, AMPD1, ARRDC5, ANKRD36BP2, C17orf96, WNT9A, FAM101A, and HSD17B6 also play significant roles in tuberculosis pathogenesis and are potential candidate therapeutic targets, warranting further investigation in future studies.

This study integrates Poly(A)-seq and RNA-seq to uncover a previously unrecognized layer of post-transcriptional regulation in TB granulomas, demonstrating that poly(A) tail length dynamically modulates gene expression in response to Mtb infection. While aligning with prior transcriptomic findings of upregulated immune activation (e.g., innate immunity, inflammation) and metabolic reprogramming (e.g., lipid/ion transport) pathways in TB granulomas, our work uniquely links these transcriptional changes to poly(A) tail dynamics, revealing 692 genes—including 22 immune/inflammation-related genes such as CD79A and CHI3L1—with significant tail elongation that likely enhances mRNA stability and translation efficiency, thereby amplifying antibacterial responses while potentially contributing to immunopathology. By focusing on poly(A) tails as a regulatory hub, the study fills a critical gap in existing literature, as post-transcriptional regulation via tail length has been entirely unaddressed in TB granuloma research. Functional enrichment analyses highlight associations with phosphorylation (a key Mtb survival mechanism) and G protein-coupled receptor signaling (implicated in host lipid metabolism hijacking), underscoring the role of post-transcriptional mechanisms in driving granuloma-specific immune and metabolic reprogramming. Notably, CHI3L1 exemplifies this interplay, with elongated tails and upregulated expression offering a novel mechanism for its known roles in macrophage polarization and inflammation during TB. While the study’s small sample size and lack of functional validation (e.g., CRISPR-mediated tail modification assays) represent limitations, the findings emphasize the need for species-specific validation in non-human primate models to address conserved immune pathways alongside granuloma architecture variations. The identified 22 genes with coordinated poly(A) tail and expression changes present promising candidate biomarkers or potential therapeutic targets for host-directed therapy (HDT), though mechanistic clarification of causality—such as whether CHI3L1 tail elongation directly enhances bactericidal activity—requires further *in vitro*/*in vivo* investigation. Collectively, this work establishes poly(A) tail length as a critical modulator of TB granuloma biology, extending traditional transcriptional frameworks and providing a foundation for translating post-transcriptional regulatory insights into innovative HDT strategies (40–42).

Against this backdrop, our study focuses on genes such as CHI3L1, which may play a pivotal role in modulating these interactions within the granuloma microenvironment. CHI3L1, also known as chitinase 3-like 1, has been implicated in various inflammatory and immune-related processes (43). In the context of TB, its potential role in shaping the granuloma and influencing immune cell functions has not been fully explored. While our study has shed some light on its association with poly(A) tail dynamics and gene expression in TB granulomas, further investigation is warranted; it might be a pilot study identifying regulatory patterns for future confirmation.

### Limitation

Our study has several notable limitations that should be addressed. First, the small sample size (*n* = 3) without technical replicates, primarily constrained by the clinical scarcity and procurement challenges of surgically resected TB granuloma tissues, may compromise the generalizability of our findings. This limitation hinders our ability to fully capture the complexity of the relationship between poly(A) tail length and transcriptional regulation, as identified in the current analysis. Additionally, while we have characterized associations between poly(A) tail dynamics and gene expression, functional validation through *in vivo* or animal models is essential to mechanistically link poly(A) tail regulation to immune responses in TB granulomas. Furthermore, the identified biomarkers require prospective cohort studies with clinical samples to assess their relevance to TB pathogenesis, as our current cross-sectional design cannot establish their utility in disease manifestation. These limitations highlight the need for future research with larger cohorts, technical replicates, and functional assays to validate our observations and translate findings into broader clinical contexts.

## SUPPLEMENTAL MATERIAL

Fig. S1 spectrum.02825-24-s0001.tif Histological lesions.

Fig. S2 spectrum.02825-24-s0002.tif Histological lesions.

Fig. S3 spectrum.02825-24-s0003.tif Histological lesions.

Supplemental material spectrum.02825-24-s0004.docx Granuloma lesions, patient base line information, and detailed DEGs of 22 genes.

Table S1 spectrum.02825-24-s0005.xlsx Base line characteristics of 3 TB patients.

Table S2 spectrum.02825-24-s0006.xlsx The detailed information of 22 selected DEGs in TB/Ctrl groups.

ASM does not own the copyrights to Supplemental Material that may be linked to, or accessed through, an article. The authors have granted ASM a non-exclusive, world-wide license to publish the Supplemental Material files. Please contact the corresponding author directly for reuse.
