# Supplementary figures and images for: Integrative analysis of Poly(A)-seq and RNA-seq reveals transcriptional regulation of poly(A) tail length in tuberculosis
Source: Microbiol Spectr. 2026 Feb 27;14(4):e02825-24. doi: 10.1128/spectrum.02825-24 (PMC13055295; doi:10.1128/spectrum.02825-24)

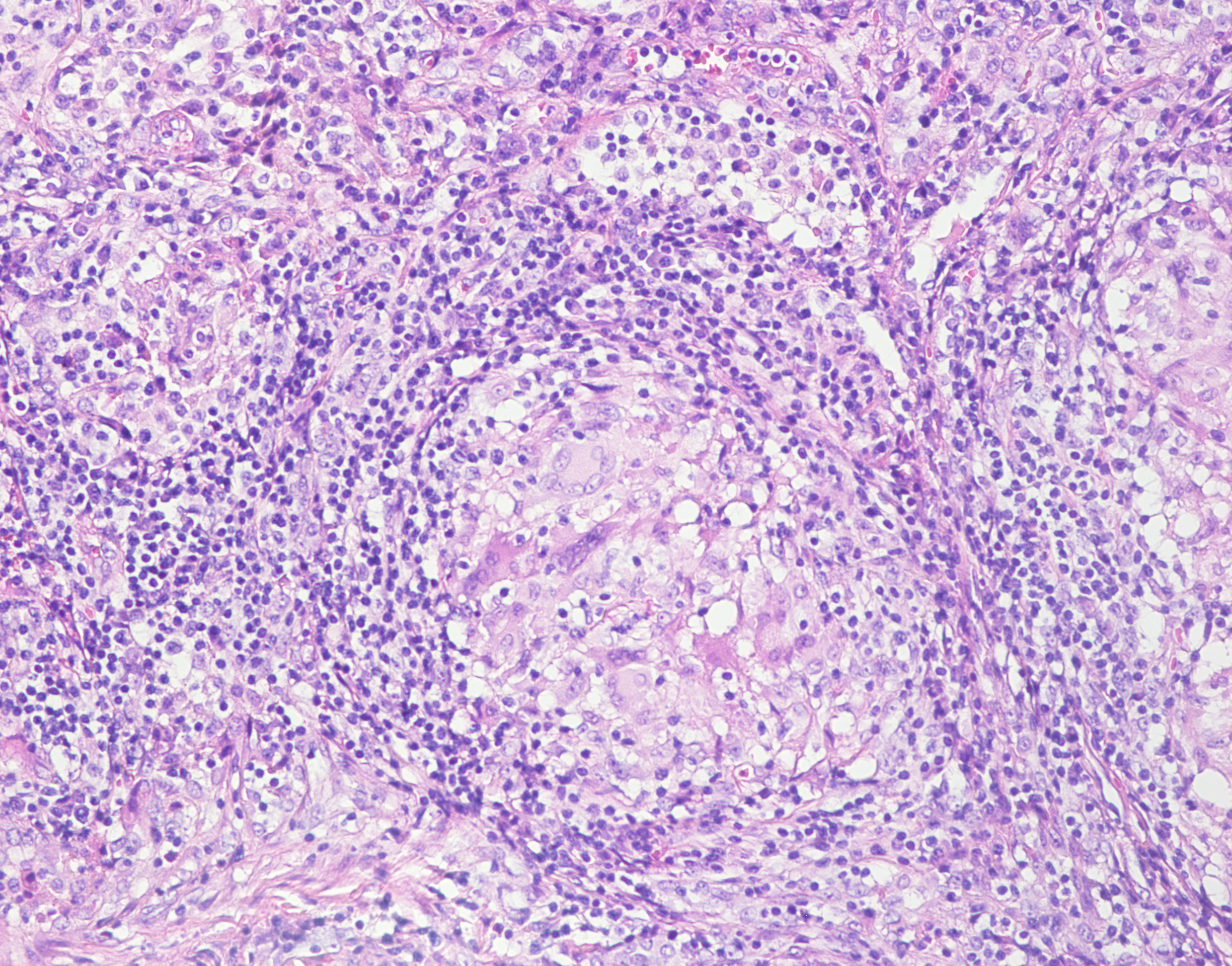

Supplement: Fig. S1 — Histological lesions. [file spectrum.02825-24-s0001.tif]

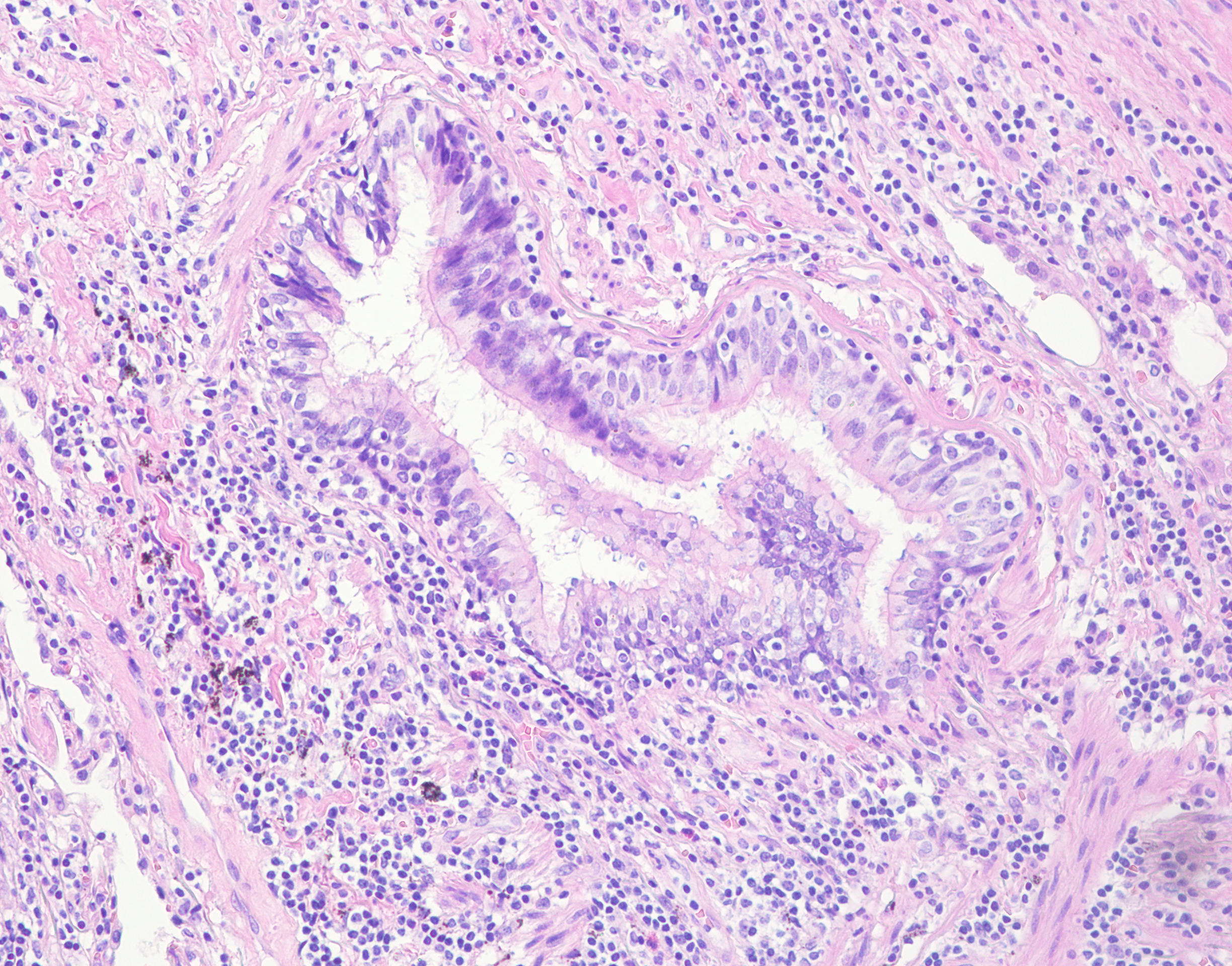

Supplement: Fig. S2 — Histological lesions. [file spectrum.02825-24-s0002.tif]

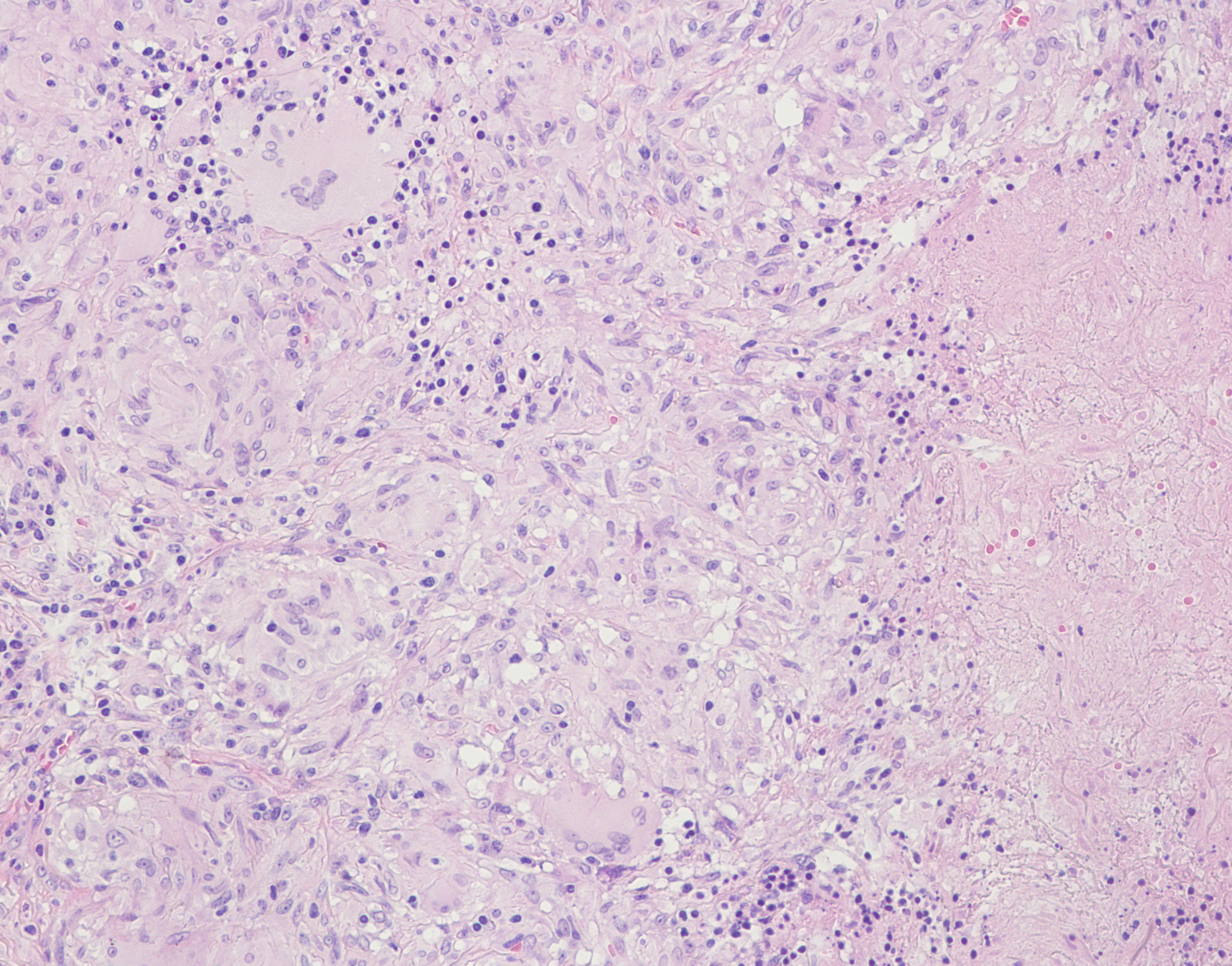

Supplement: Fig. S3 — Histological lesions. [file spectrum.02825-24-s0003.tif]
