# Supplementary material for: Integrative analysis of Poly(A)-seq and RNA-seq reveals transcriptional regulation of poly(A) tail length in tuberculosis
Source: Microbiol Spectr. 2026 Feb 27;14(4):e02825-24. doi: 10.1128/spectrum.02825-24 (PMC13055295; doi:10.1128/spectrum.02825-24)
Supplement: Supplemental material — Granuloma lesions, patient base line information, and detailed DEGs of 22 genes. [file spectrum.02825-24-s0004.docx]

**Supplementary information**

**List of supplementary figure legends**

Figure S1-3：Hematoxylin and eosin (H&E) -stained sections of granulomatous lesions with necrosis, morphologically consistent with tuberculosis, observed at 20× magnification. The images exhibit characteristic histological features, including epithelioid cells, multinucleated giant cells, and regions of caseous necrosis surrounded by inflammatory cells. These findings are typical of tuberculous granulomas, supporting the morphological diagnosis of tuberculosis.

**Supplementary table legends**

**Supplementary table 1**. Base line characteristics of three TB patients. **Supplementary table 2**. The detailed information of 22 selected DEGs in MTB/Ctrl groups.
